# Phillyrin reduces ROS production to alleviate the progression of intervertebral disc degeneration by inhibiting NF-κB pathway

**DOI:** 10.1186/s13018-024-04695-y

**Published:** 2024-05-22

**Authors:** Enming Chen, Ming Li, Zhuangyao Liao, Dengbo Yao, Yuxi Li, Lin Huang

**Affiliations:** 1 Department of Orthopedics, Sun Yat-sen Memorial Hospital, Sun Yat-sen University, 107 Yanjiang West Road, Guangzhou, 510120 China; 2 Department of Orthopedics, the Eighth Affiliated Hospital of Sun Yat-sen University, Sun Yat- sen University, Shenzhen, China

**Keywords:** Phillyrin, Intervertebral disc degeneration, Inflammation, Apoptosis, Oxidative stress, NF-κB, MAPK

## Abstract

**Background:**

Intervertebral disc degeneration (IDD) is an increasingly important cause of low back pain (LBP) that results in substantial health and economic burdens. Inflammatory pathway activation and the production of reactive oxygen species (ROS) play vital roles in the progression of IDD. Several studies have suggested that phillyrin has a protective role and inhibits inflammation and the production of ROS. However, the role of phillyrin in IDD has not been confirmed.

**Purpose:**

The purpose of this study was to investigate the role of phillyrin in IDD and its mechanisms.

**Study design:**

To establish IDD models in vivo, ex-vivo, and in vitro to verify the function of phillyrin in IDD.

**Method:**

The effects of phillyrin on extracellular matrix (ECM) degeneration, inflammation, and oxidation in nucleus pulposus (NP) cells were assessed using immunoblotting and immunofluorescence analysis. Additionally, the impact of phillyrin administration on acupuncture-mediated intervertebral disc degeneration (IDD) in rats was evaluated using various techniques such as MRI, HE staining, S-O staining, and immunohistochemistry (IHC).

**Result:**

Pretreatment with phillyrin significantly inhibited the IL-1β-mediated reduction in the degeneration of ECM and apoptosis by alleviating activation of the NF-κB inflammatory pathway and the generation of ROS. In addition, in vivo and ex-vivo experiments verified the protective effect of phillyrin against IDD.

**Conclusion:**

Phillyrin can attenuate the progression of IDD by reducing ROS production and activating inflammatory pathways.

## Introduction

LBP is a common disabling factor in the world and places substantial health and economic burdens on society. Statistically, 80% of adults have suffered from low back pain in their lifetime [1]. In clinical settings, 40% of LBP is attributed to IDD, a degenerative musculoskeletal condition associated with the spine. IDD may be a secondary consequence of various conditions, including spinal canal stenosis, lumbar disc herniation, and lumbar spondylolisthesis [2, 3]. Currently, common treatments for IDD are physical therapy, nonsteroidal anti-inflammatory drug treatment, and surgical treatment. However, these treatments only relieve symptoms without slowing the progression of IDD. Therefore, it is imperative to develop medications aimed at addressing the underlying causes of IDD to restore the integrity of the intervertebral disc (IVD).The IVD is comprised of three essential components: the nucleus pulposus (NP), annulus fibrosus (AF), and cartilaginous end plate (CEP). Among these components, the NP is primarily associated with the IVD, encompassing nucleus pulposus cells (NPCs) and extracellular matrix (ECM). A reduction in NPCs and the degradation of ECM lead to the progression of IDD. NPC apoptosis is significantly related to a reduction in NPC numbers [4, 5]. A reduction in the production of ECM, specifically Collagen2 and Aggrecan, coupled with an elevation in the expression levels of MMPs (MMP3, MMP9, MMP13) and ADAMTSs (ADAMTS5) lead to a reduction in ECM.

Accumulating evidence indicates that activation of the inflammatory pathway and overproduction of ROS play critical roles in the pathogenesis of IDD. Recently, activation of inflammatory pathways has been identified as a key regulator of NPC catabolism leading to IDD progression [6]. Jiang et al. found that excessive ROS levels disrupt disc matrix homeostasis, and regulate matrix metabolism, inflammation, apoptosis, and autophagy, resulting in the disruption of ECM and IDD [7, 8]. Therefore, studying the role of inflammatory pathways and ROS may be an important approach for alleviating the progression of IDD.

Forsythia suspensa (Thunb.) Vahl, a plant found in China, Korea, and Japan, is prevalent in these regions. It is important to note that Forsythia fructus possesses remarkable anti-allergy and anti-inflammatory properties [9, 10]. Phillyrin, one of the major constituents of Forsythia suspensa, exhibits antioxidant, anti-inflammatory and anti-obesity effects [11–13]. It is unknown whether phillyrin can affect the progression of IDD and the specific mechanism. Considering the significant impact of inflammation and oxidative stress on intervertebral disc degeneration, our study aimed to investigate the potential protective effects of phillyrin in this process. Our study showed that phillyrin could inhibit the occurrence and progression of IDD by alleviating matrix degradation and reducing ROS and apoptosis levels in inflammatory environments. Our study provides a theoretical basis for selecting targets for IDD.

## Methods

### Cell culture

The lumbar IVD tissues of 8-week-old male rats were obtained and processed for further experiments. The tissues were initially treated with 0.2% pronase at 37 °C for 1 h, followed by 15 min of exposure to 2.5% collagenase II at the same temperature. The NPCs were then collected from the digested tissues and cultured in DMEM supplemented with 10% FBS and 1% antibiotics. The cells were maintained at 37 °C, 5% CO2, and 20% O2, with the medium being changed every two days until they reached 80–90% confluence^12,13^.

### Cell viability analysis

We seeded 96-well plates with 2 × 10^3^ cells per well for 24 h before adding increasing concentrations of phillyrin (0, 10, 20, 40, 80, 160 and 320 µM in DMSO; Med Chem Express, MCE, China). Following phillyrin treatment, the cells were cultured in 10 µL of CCK-8 reagent(Med Chem Express, MCE, China) in fresh complete media for 1 h at 37 °C. In the simulated control, untreated cells were compared to cells in control medium and treated with CCK-8 reagents in the blank control. In this study, optical density readers were used to measure the absorbance at 450 nm.

### Western blot analysis

We utilized RIPA buffer containing 1% proteinase inhibitor and 1% phosphotransferase inhibitor (Cwbio, Jiangsu, China) to lyse the NPC samples. Total protein extraction was then carried out through centrifugation.

Subsequently, western blot analysis was performed on each sample, utilizing 20 µl of protein. To account for variations in protein weights, the preboiled protein samples and loading buffer went through electrophoresis for 90 min on a 10% or 12% SDS-PAGE gel. The gel particles were transferred onto a PVDF membrane (Millipore, Billerica, MA, USA). The membranes were cut horizontally to identify proteins of various sizes. For antibody detection, we employed the following antibodies at the indicated dilutions: anti-MMP3 (1:1000), anti-MMP9 (1:1000) from Abcam; anti-p38 (1:1000), anti-p-p38 (1:1000) from CST; anti-MMP13 (1:1000), anti-Aggrecan (1:1000), anti-Bcl2 (1:1000), anti-Bax (1:1000), anti-Caspase-3(1:1000), anti-p65 (1:1000), and anti-p-p65 (1:1000) from Immunoway; anti-GAPDH (1:5000) and anti-Tubulin (1:5000) from Cwbio.

Following incubation with the primary antibodies, the membranes were washed using TBST and subsequently incubated with secondary antibodies for one hour. Following three rounds of washing, the membranes were incubated with secondary antibodies for one hour. Signals were detected and analyzed using an ECL imager (Syngene G: BOX ChemiXT4, United Kingdom).

### Immunofluorescence analysis

The cells underwent three washes with PBS, were then fixed with 4% paraformaldehyde, and washed with 0.5% Triton X-100 for 5 min. To prevent any nonspecific binding, a solution of bovine serum albumin (1%) was applied and left for 1 h at 37 °C. Following this step, the cells were washed with PBS and subsequently incubated with primary antibodies overnight at 4 °C. The cells were subjected to three consecutive washes with PBS, after primary antibody incubation, then incubated with the secondary antibody (Alexa Fluor® 488/594 conjugated, at a concentration of 1:100) for 1 h. Additionally, DAPI staining was performed for 5 min at 37 °C. A microscope, specifically the Olympus BX63 (NY, USA), was used to capture images after three further washes with PBS.

### ROS flow cytometry

In order to assess the presence of intracellular reactive oxygen species (ROS) at the individual cell level, we utilized a staining technique. Specifically, we treated the cells with a solution containing 10µM DCFH-DA in DMEM lacking fetal bovine serum (FBS) for 30 min. This treatment occurred at 37℃, within an environment enriched with 5% CO2. Subsequently, we detached the cells by applying a trypsin solution and subjected them to centrifugation in 1.5 ml tubes at a force of 500 × g for 4 min. Following the removal of the supernatant, we resuspended the resulting cell pellet in PBS (phosphate-buffered saline). By employing a specialized instrument, we collected flow cytometry data, which was then analyzed utilizing Flow Jo software to determine the geometric mean fluorescence intensity. For comparison, we included unstained and untreated samples as controls in our analysis.

### Surgical procedures

Approval for all animal experiments conducted in this study was obtained from the Institutional Animal Care and Use Committee (SYSU-2022-G0104) at Sun Yat-Sen University. Sprague–Dawley rats, were procured from Sun Yat-sen University’s Laboratory Animal Center, and they were free access to food and water for a week before to the experiment. They were randomly divided into three distinct groups, namely the control, IDD, and phillyrin groups. To ensure compliance with ethical guidelines, rats in the IDD and phillyrin groups were administered 2% pentobarbital (50 mg/kg) for anesthesia and subsequently punctured using a 21G needle through the skin. Following a 30-second interval, 10µM of phillyrin was precisely injected into the discs of rats within the phillyrin group, while an equal volume of DMSO was injected into the discs of rats within the IDD group. The experiment was conducted in a controlled environment, ensuring the rats’ well-being by providing them with ample water and food and maintaining suitable temperature, humidity, and light conditions.

### Effect of phillyrin on an ex vivo IVD culture model

The IVDs, having intact endplates, were extracted from the rats and subjected to culture in DMEM supplemented with 10% FBS and 1% penicillin/streptomycin. As per prior investigations, the specimens were cultured under conditions of 37 °C and 5% oxygen while maintaining optimal humidity levels [14]. During the culture process, IL-1β and phillyrin were administered to the IVDs. The medium was routinely replaced every 48 hours.

### Magnetic resonance imaging method

4 weeks after surgery, we utilized MRI to investigate disc degeneration in rats. Intraperitoneal injection of an overdose of pentobarbital (50 mg/kg) was employed to euthanize each rat. The rat tails were then examined using a 3.0 T clinical magnet (Philips Intera Achieva 3.0MR) to detect the signal and structures in sagittal T2-weighted images [15]. We evaluated the degeneration of intervertebral discs according to the system [16, 17].

### Histological and immunohistochemical (IHC) staining

The specimens were preserved by immersing them in a solution of 4% paraformaldehyde for 48 h. Following this, a decalcification process lasting over 30 days was performed, after which the specimens were embedded in paraffin. In order to evaluate disc degeneration, the sections were subjected to routine staining techniques utilizing Haematoxylin-eosin stain and Safranin O-fast green-staining. Subsequently, the sections underwent incubation with primary antibodies targeting MMP3 (1:200) and BAX (1:200), and the disc samples were then exposed to secondary antibodies. ImageJ software was used in visualizing the images, as well as calculating the histology scores based on an established grading system [18, 19].

### Statistical analysis

We repeated each experiment at least three times, and expressed the results as the mean ± SD. Statistical analysis was conducted using GraphPad Prism 8 software (La Jolla, CA, USA). The Student’s t-test and two-way ANOVA were employed to assess statistical significance, with P values below 0.05 considered statistically significant.

## Results

### Effect of phillyrin on NPC viability

**Fig. 1:**
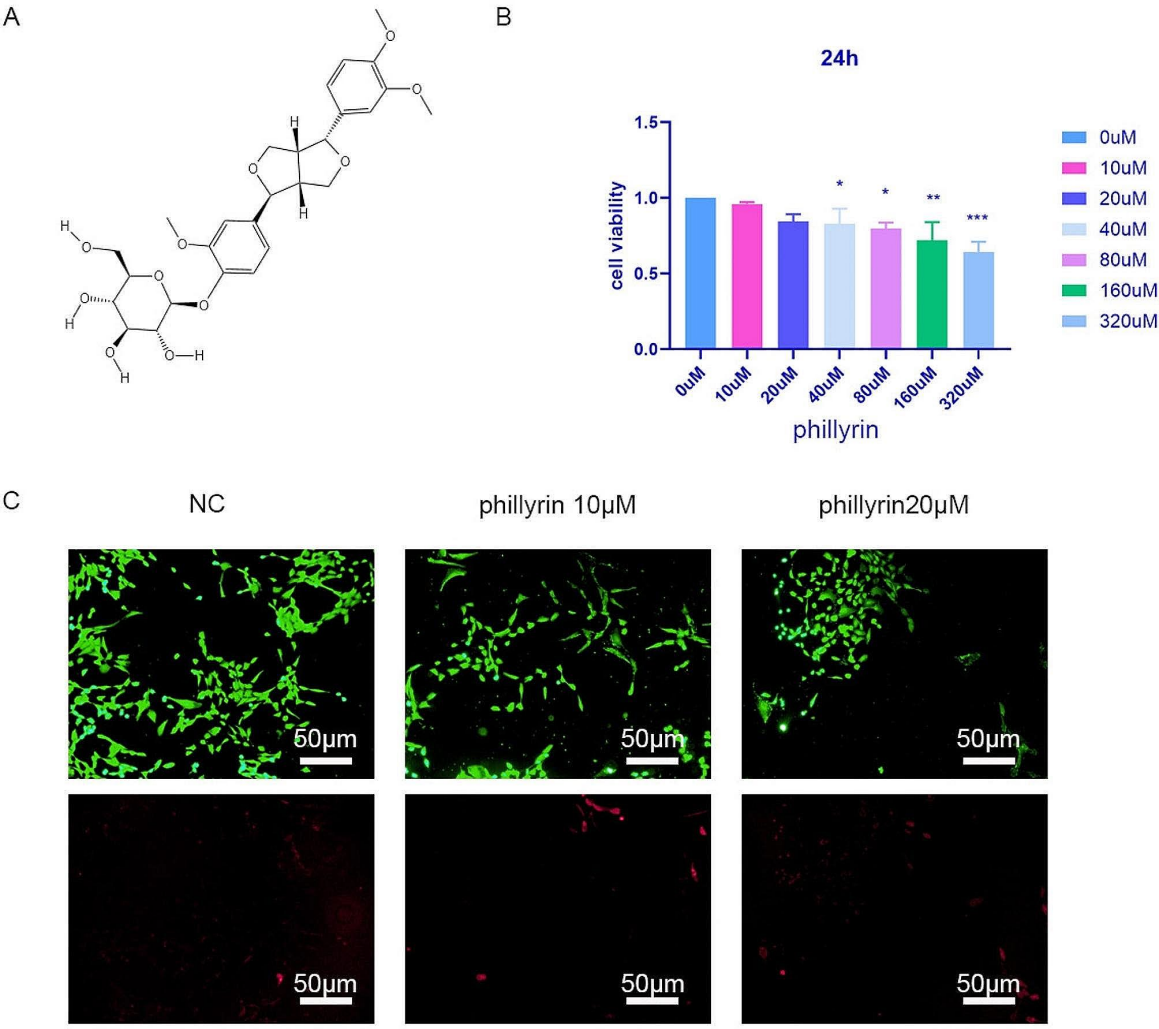
**Effect of phillyrin on NPC viability** (**A**) chemical structure formula of phillyrin. (**B**) viability of rat NPCs cultured with 0 ~ 320 µM phillyrin for 24 h. (**C**) LIVE/DEAD cell staining of NPCs treated with or without phillyrin for 24 h

The structural formula of phillyrin is shown in Fig. 1A. First, we used the Cell Counting Kit-8 (CCK8) viability assay to detect the effect of phillyrin on the viability of NPCs. Based on the findings, it was observed that the viability of NPC was unaffected when treated with phillyrin at concentrations below 20 µM for 24 h compared to the negative control group (Fig. 1B). Thus, 10 and 20 µM were selected for the low and high doses, respectively. The effects of 10 and 20 µM phillyrin on NPC proliferation were evaluated by living/dead staining. Green staining represents living cells, while red staining represents dead cells. Compared with untreated controls, treatment with 10 and 20 µM phillyrin did not affect cell proliferation (Fig. 1C). Hence, 10 and 20 µM were chosen as the doses in the following experiments.

### Phillyrin alleviates IL-1β-induced ECM degradation in NPCs

**Fig. 2:**
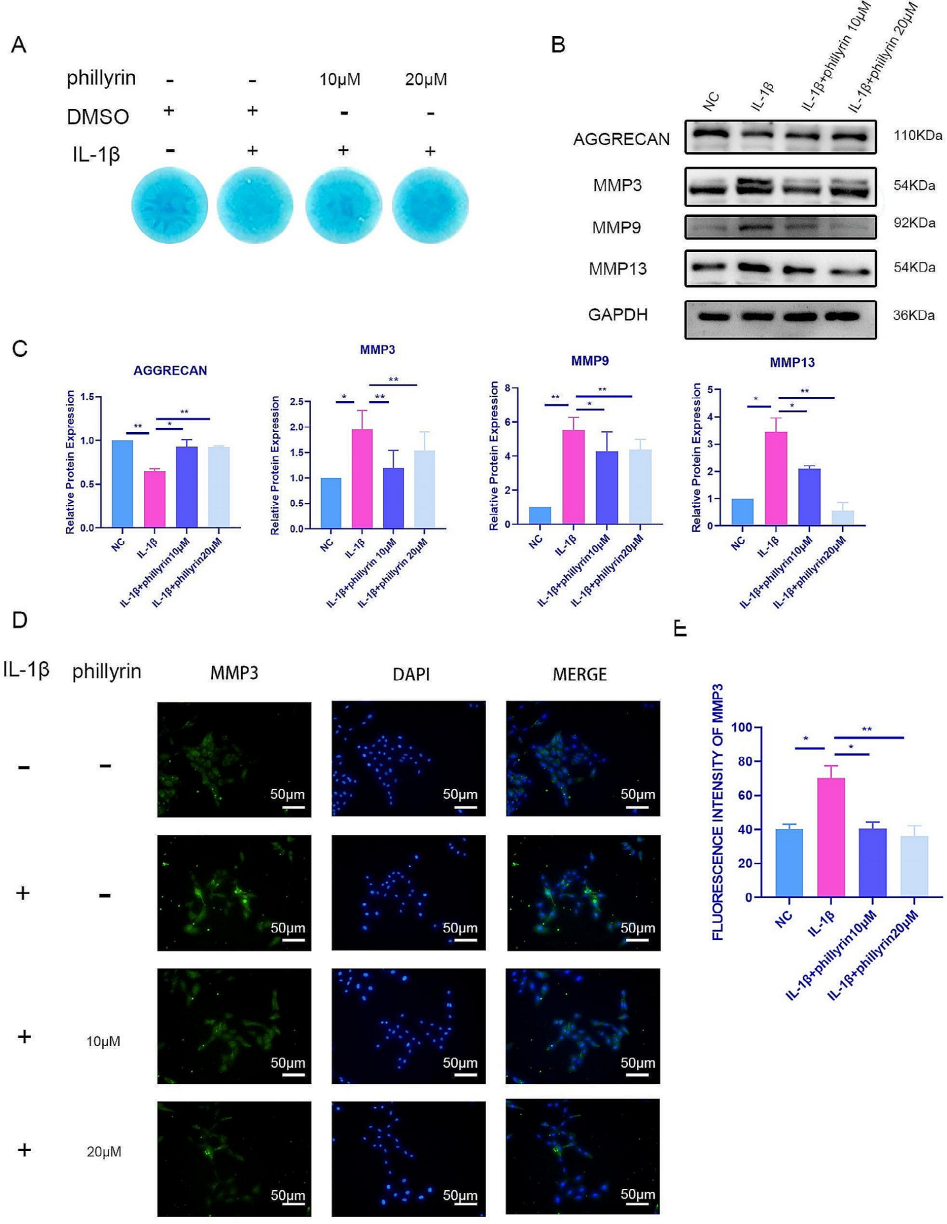
**Phillyrin alleviates IL-1β-induced NPC extracellular matrix degradation.** (**A**) high-density culture of NPCs treated or not with 40ng/ml IL-1β for 5 days after pretreat with or without phillyrin for 12 h. (**B**) AGGRECAN, MMP3, MMP9, MMP13 protein expression of NPCs treated or not with 40 ng/ml IL-1β for 48 h after pretreat with or without phillyrin for 2 h. (**C**) Quantification of specific signal intensities. GAPDH was used as the loading control. Values are presented as mean ± standard deviation (SD) for three independent experiments. *p < 0.05, **p < 0.01, ***p < 0.001. (**D**) Immunofluorescence images ( 20 X ) showing MMP3 staining of NPCs treated or not with 40ng/ml IL-1β for 48 h after pretreat with or without phillyrin for 2 h. (**E**) ImageJ was used to analyze the immunofluorescence intensity of MMP3. The data are presented as the mean ± SD (*P < 0.05; **P < 0.01; ***P < 0.001)

The amount of ECM secreted by NPCs is critical to the progression of IDD. To assess the effect of phillyrin on alleviating the reduction in ECM caused by IL-1β, we introduced phillyrin to a high-density culture of nucleus puposus cells (NPCs). IL-1β exposure reduced Alcian staining compared with that in the untreated group and phillyrin pretreatment significantly rescued staining (Fig. 2A). Decrease in ECM (aggrecan) and increases in matrix-degrading proteases (MMPs and aggrecanses) are catabolic and anabolic markers of IDD pathogenesis [20]. We further examined whether phillyrin could alleviate the changes in IDD hallmarks caused by IL-1β. NPCs were pretreated with phillyrin (10 µM and 20 µM) for 2 h and then stimulated with 40 ng/ml IL-1β for 48 h. IL-1β stimulation decreased the protein expression of aggrecan in NPCs and increased MMP3, MMP9, and MMP13 expression, which are related to the progression of IDD. Pretreatment with phillyrin significantly reversed the protein expression of these factors (Fig. 2B, C). Similarly, immunofluorescence analysis of MMP3 showed that phillyrin could ameliorate the increase in MMP3 protein expression caused by IL-1β in NPCs (Fig. 2D, E). Consequently, phillyrin inhibited ECM degradation in NPCs caused by IL-1β.

### Phillyrin attenuates IL-1β-induced apoptosis in NPCs

**Fig. 3:**
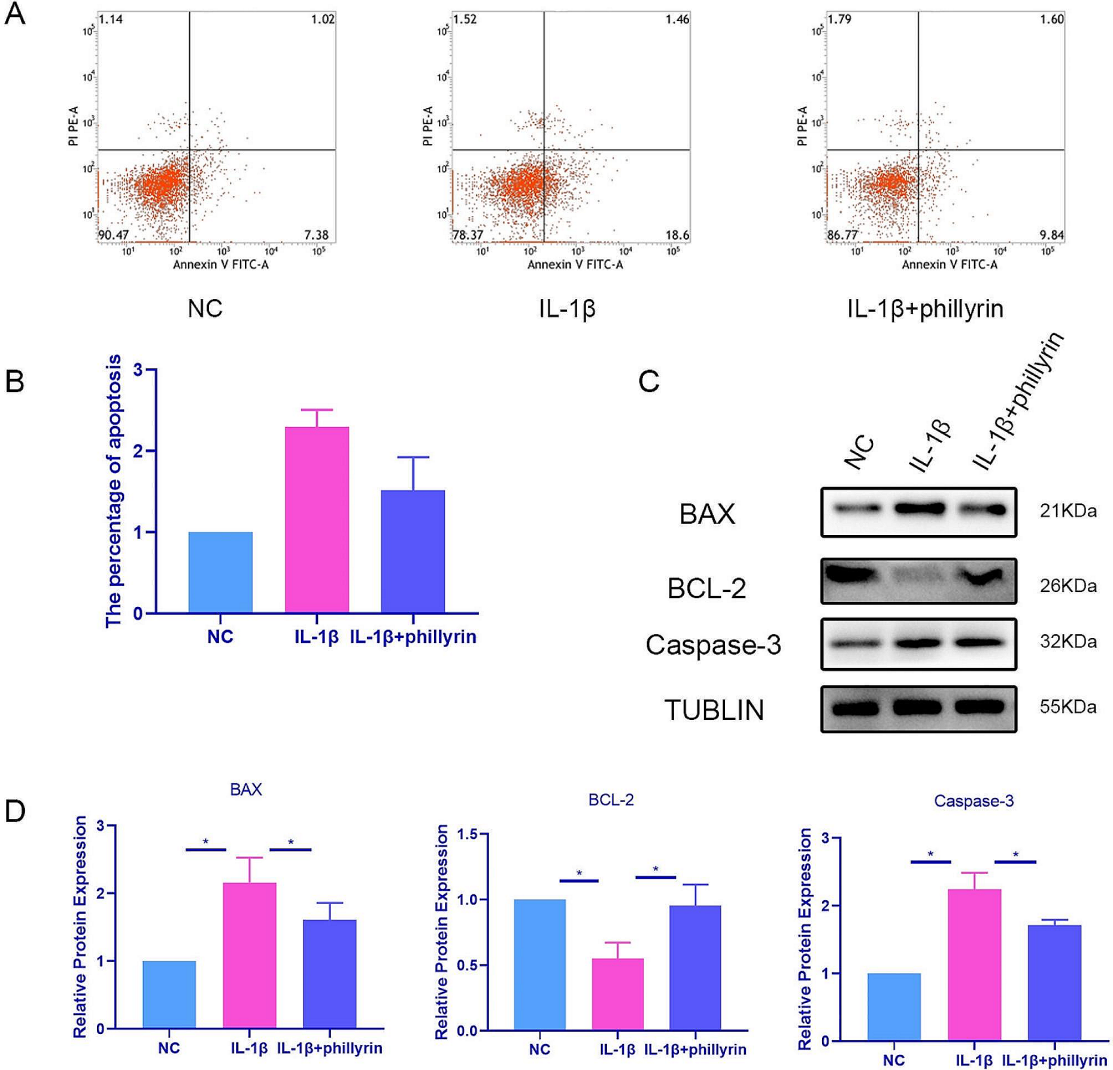
**Phillyrin attenuates IL-1β-induced apoptosis in NPCs.** (**A**) Apoptotic cell rates were detected by a FITC annexin V-FITC/PI apoptosis kit, and analyzed by flow cytometry of NPCs treated or not with 40 ng/ml IL-1β for 12 h after pretreat with or without phillyrin for 2 h. (**B**) quantification of apoptosis. (**C**) BAX, BCL-2, Caspase-3 protein expression of NPCs treated or not with 40 ng/ml IL-1β for 48 h after pretreat with or without phillyrin for 2 h. (**D**) Quantification of specific signal intensities. TUBLIN was used as loading control. Values are presented as mean ± standard deviation (SD) for three independent experiments. *p < 0.05, **p < 0.01, ***p < 0.001

Apoptosis plays a crucial role in the development of IDD, and we set out to examine the impact of phillyrin on the apoptosis induced by IL-1β in NPCs. Through flow cytometry, we observed that the percentage of NPCs undergoing apoptosis after being exposed to 40 ng/ml IL-1β for 24 h was higher compared to the untreated group. However, when NPCs were pre-treated with phillyrin, this increase in apoptosis was effectively prevented (Fig. 3A, B). Analysis using Western blot revealed that the levels of BAX and Caspase-3 increased while BCL-2 decreased upon treatment with IL-1β, indicating stimulation of NPC apoptosis and progression of IDD. Nevertheless, this effect was counteracted by the presence of phillyrin, which corresponded with the findings from the flow cytometry (Fig. 3C, D). Hence, phillyrin exhibits a mitigating effect on the IL-1β-induced apoptosis in NPCs.

### Phillyrin alleviated IL-1β-induced ECM degradation in an ex-vivo IVD culture model

**Fig. 4:**
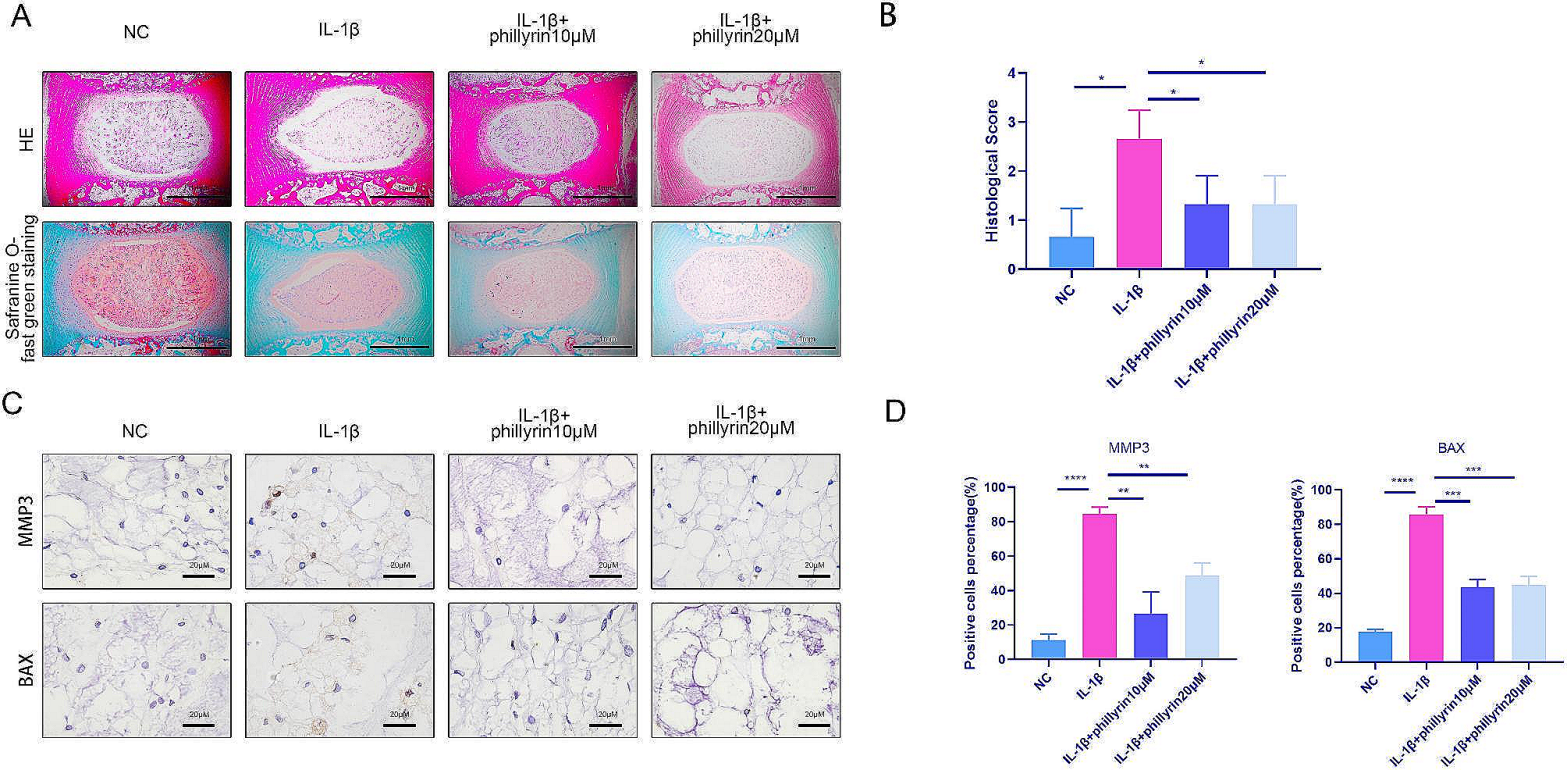
**Phillyrin alleviated IL-1β-induced ECM degradation in an ex-vivo culture model of IVD.** (**A**) Histological analysis including HE and S-O staining showed the morphology of disc organ treated or not with 40 ng/ml IL-1β for 14 d after pretreat with or without phillyrin for 2 d. (**B**) The histological grades were evaluated (*n* = 3) (**C**) Expression of MMP3 and BAX in disc organs treated or not with 40 ng/ml IL-1β for 14 d after pretreat with or without phillyrin for 2 d. (**D**) The positive cells percentage was evaluated

Since the protective effect of phillyrin on NPCs in vitro has been conclusively demonstrated by these findings, itbecomes imperative to investigate whether phillyrin offers protection to the nucleus pulposus as IDD progresses in an ex-vivo IVD culture model. IVDs with intact end plates were obtained from rats and subjected to culture in DMEM supplemented with 10% FBS and 1% penicillin/streptomycin. As shown in Fig. 4A, B and S-O and HE staining showed that the IL-1β group had more tear fluid and smaller nucleus pulposus tissue areas than the other groups, but phillyrin reversed this trend. Moreover, we used IHC to detect the expression of MMP3 and BAX. IHC staining showed that BAX and MMP3 levels were higher in the IL-1β group than in the untreated group, and phillyrin inhibited these effects (Fig. 4C, D). In conclusion, these results indicate that phillyrin alleviates the degeneration of isolated IVDs.

### Phillyrin inhibits ROS generation and NF-κB pathway activation in IL-1β-treated NPCs

**Fig. 5:**
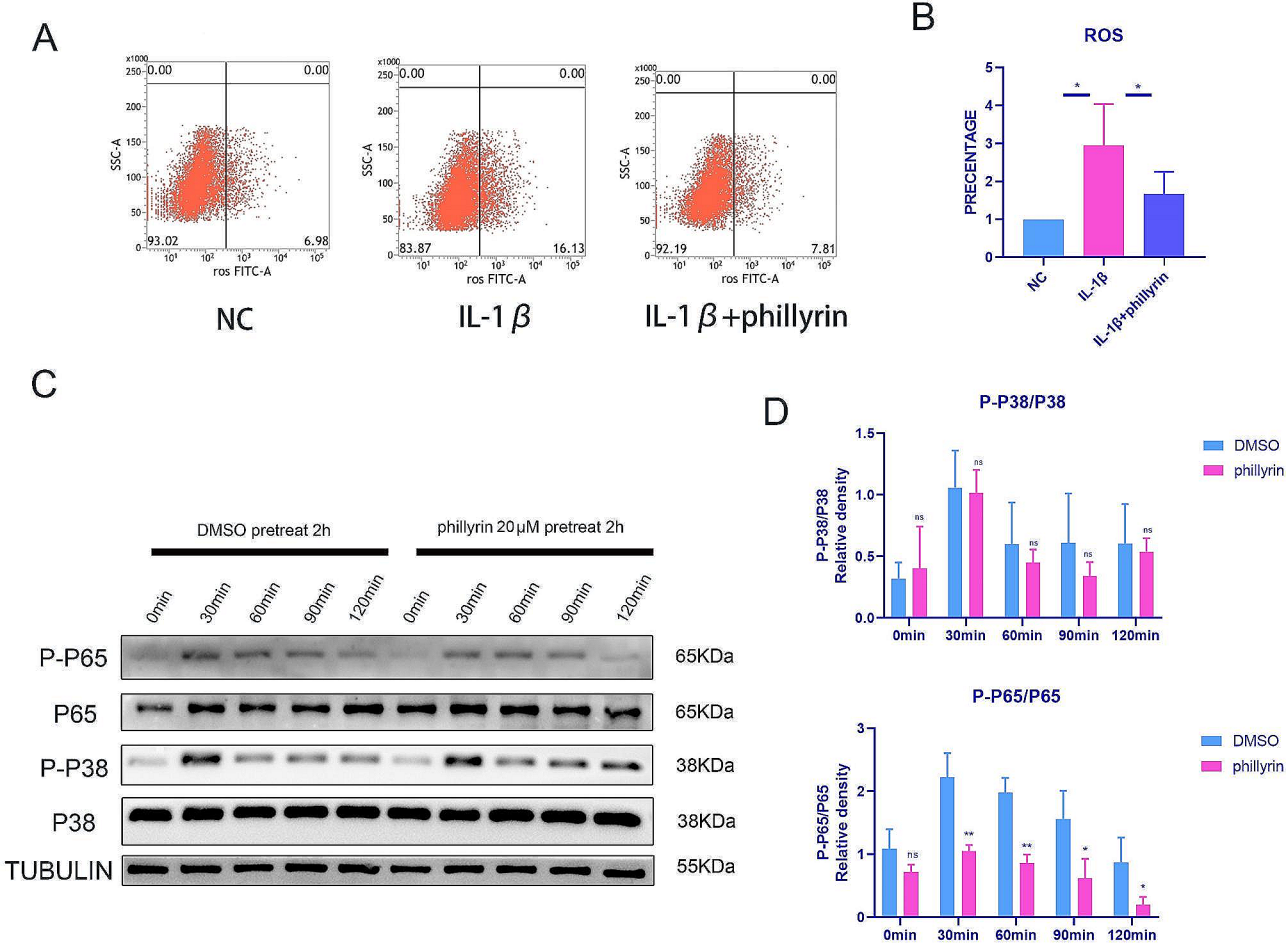
**Phillyrin inhibits ROS generation and NF-κB pathway activation in IL-1β-treated NPCs.** (**A**) ROS staining of NPCs treated or not 40 ng/ml IL-1β for 12 h after pretreat with or without phillyrin for 2 h. (**B**) Quantification of ROS. (**C**) Western blot showing the phosphorylated of p65 of NPCs treated or not with 40 ng/ml IL-1β for 2 h after pretreat with or without phillyrin for 2 h. (**D**)Quantification of specific signal intensities. Values are presented as mean ± standard deviation (SD) for three independent experiments. *p < 0.05, **p < 0.01, ***p < 0.001

Next, we explored the molecular mechanism by which phillyrin alleviates the progression of IDD. Previous research has established IL-1β activates NF-κB, MAPK kinase and ROS generation to promote ECM degradation and NPCs apoptosis, And phillyrin has antioxidant and anti-inflammatory properties. Therefore, we conducted experiment to investigate whether phillyrin affected IL-1β-induced oxidative stress in NPCs. Flow cytometry was used to measure ROS, which determined the extent of oxidant stress in NPCs. We found the presence of IL-1β led to a notable increase in the production of intracellular ROS. However, when the cells were pre-treated with phillyrin, a significant reduction in the generation of intracellular ROS was observed (Fig. 5A, B). This demonstrates the ability of phillyrin to effectively alleviate IL-1β-induced oxidative stress by inhibiting the formation of ROS in NPCs. We also conducted an experiment to evaluate the levels of phosphorylated P65 and P38 in NPCs subjected to various treatments, and demonstrated that the exposure to IL-1β resulted in the phosphorylation of P65 and P38 after 30 min. Conversely, when pretreated with phillyrin for 2 h, a noteworthy inhibition of P65 phosphorylation was observed, while P38 remained unaffected (Fig. 5C, D). In conclusion, these findings strongly suggest that phillyrin hinders the IL-1β-triggered activation of NF-κB and the subsequent generation of ROS.

### Phillyrin alleviated the progression of intervertebral disc degeneration in vivo

**Fig. 6:**
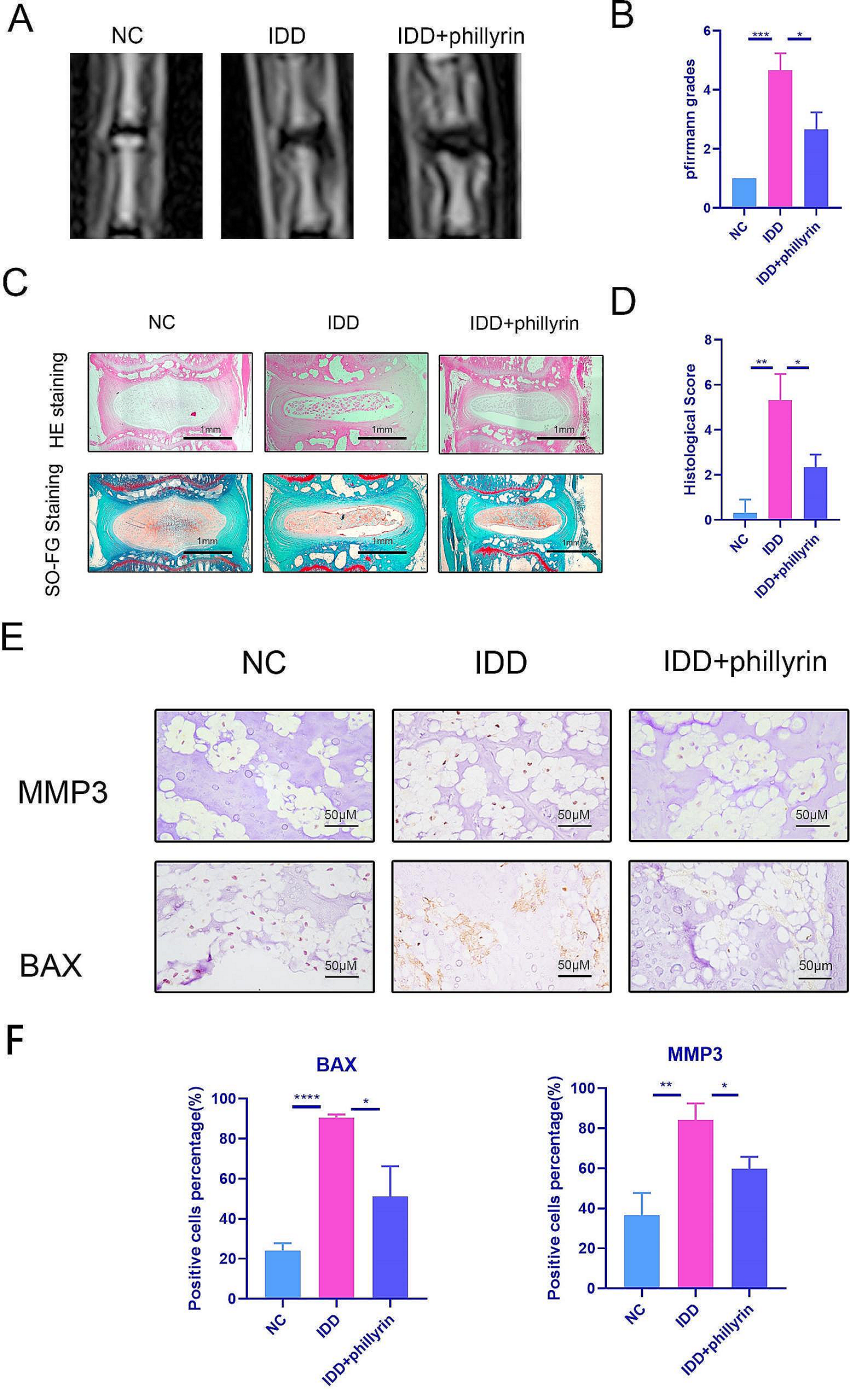
**Phillyrin alleviated the progression of IDD in vivo.** (**A**) Representative IVD revealed by MRI on T2-weighted images. (**B**) Pfirrmann score of caudal MRI images of rats after surgery and injection with phillyrin (**C**) HE and S-O staining of IVDs. (**D**) The histological grades were evaluated (*n* = 3) (**E**) IHC was used to determine the protein expression of MMP3 and BAX. (**F**) The positive cells percentage was evaluated

To conduct further research on the protective effects of phillyrin on the nucleus pulposus in living organisms, we conducted experiments on rat caudal IDD models using acupuncture. Phillyrin was administered to the rats’ intervertebral discs as a treatment for acupuncture-induced IDD. The results obtained from MRI images taken in 6th week following the procedure indicated a significant decrease in signal intensity, signifying degeneration of the intervertebral disc after acupuncture. However, the administration of phillyrin resulted in a slight decrease in signal intensity (Fig. 6A, B). To further examine the impact of phillyrin, S-O and HE staining were performed, revealing that phillyrin prevented the loss of NP phillyrin after acupuncture (Fig. 6C, D). Additionally, we investigated the expression levels of MMP3 and BAX in IVDs. As anticipated, IHC revealed an increase in MMP3 and BAX expression in IVDs after acupuncture, which was significantly mitigated by the administration of phillyrin (Fig. 6E, F). In conclusion, these findings demonstrate that phillyrin possesses the ability to impede the progression of IDD in living organisms through the inhibition of NPC apoptosis and ECM degradation.

## Discussion

LBP has brought huge medical and socioeconomic burdens to modern society, and IDD is the main cause of LBP, accounting for 40% of LBP cases. Because of its intricate pathogenesis, the primary clinical treatment approaches encompass analgesic medication, partial excision through surgery, or complete intervertebral disc (IVD) substitution. Therefore, there is an urgent need to develop highly efficient, safe, and stable drugs to improve IDD. Phillyrin has antioxidant and anti-inflammatory effects, both of which are associated with the aetiology of IDD. The data revealed a noteworthy discovery: phillyrin effectively suppressed apoptosis and oxidative stress, as well as the activation of NF-κB and matrix-degrading proteases linked to NPC degradation, induced by IL-1β. Therefore, our results suggest that phillyrin is of great value in inhibiting the development of IVDD.

Previous studies have shown that ECM degradation plays a critical role in IDD. Excessive ECM catabolism has been identified as an important phenotype for degenerative problems in the IVD [21]. An increase in ECM catabolic enzymes (MMPs and ADAMTS) and a decrease in anabolic genes (AGGRECAN) were observed in IDD [22]. In this research, we observed a significant rescue of the protein expression changes induced by IL-1β through phillyrin pretreatment. Additionally, phillyrin exhibited the capacity to alleviate NPC apoptosis triggered by IL-1β. Existing literature highlights the continual occurrence of cell apoptosis, which critically contributes to the degenerative advancement of IVD [23–25]. The treatment of NPCs with IL-1β resulted in an elevation of BAX and a reduction in BCL-2, two crucial markers of programmed cell death. When phillyrin was administered, there was a decrease in BAX levels and an increase in BCL-2 expression, aligning with the flow cytometry findings. These results indicate that the pre-administration of phillyrin effectively inhibited IL-1β-induced apoptosis in NPCs.

We further investigated the mechanism by which phillyrin inhibits IL-1β-induced apoptosis and ECM degradation in NPCs. During IDD, abnormal activation of the inflammatory pathway caused by the overexpression of IL-1β causes ECM degradation in NPCs. The mitogen-activated protein kinase (MAPK) and NF-κB pathways are master regulators of inflammation and catabolism during IDD progression. The MAPK signalling pathway appears to play a key role in regulating matrix synthesis and breakdown in IVD by altering the expression of anabolic and catabolic genes and affecting proteoglycan breakdown in IVD [26–28]. But in this study, phillyrin did not inhibit activation of the MAPK pathway, and the specific mechanism needs further study. However, the phosphorylation of P65 can be inhibited by phillyrin pretreatment, much evidence was demonstrated that activation of NF-κB in IVD promotes the progression of IDD. MMP3, MMP9, MMP13 and ADAMTS5 have been identified as NF-κB target genes in NPCs [6]. IL-1β activates NF-κB signalling through P65 phosphorylation, and inhibiting P65 phosphorylation significantly reduces IL-1β-induced increases in MMP3, MMP9, and MMP13^28^. This may be one of the reasons why phillyrin rescued IL-1β-induced upregulation of MMP3, MMP9, and MMP13 expression. Furthermore, NF-κB activation in NPCs correlates with the accumulation of ROS [29]. This study shows that phillyrin has antioxidant effects on NPCs, and the inhibitory effect of phillyrin on IL-1β-induced P65 phosphorylation may be due to the inhibition of ROS accumulation. ROS are unstable, highly reactive compounds that are by-products of cellular oxidative metabolism and can cause oxidative damage to DNA, lipids, and proteins [32].Degenerated IVDs produce excessive ROS, and the occurrence and development of IDD are closely related to ROS and oxidative stress [30, 31]. Too much ROS utimately lead to cell damage and death [33]. Elevated ROS levels in NPCs activate senescence signalling pathways and inhibit cell proliferation, thereby increasing IDD [34–36]. Our experimental results showed that pretreatment with phillyrin significantly alleviated the generation of ROS induced by IL-1β, which is consistent with the antioxidant properties of phillyrin demonstrated in previous studies [37].

Our findings reveal the impact of phillyrin on the advancement of IDD. The influence of phillyrin on IL-1β-induced ECM degradation and NPC apoptosis is showcased through its ability to suppress NF-κB activation and excessive ROS generation. Consequently, phillyrin has potential as a therapeutic agent for alleviating IDD.

However, this study has some limitations. First, we did not address the target of phillyrin. Second, it remains uncertain whether phillyrin’s inhibitory impact on the activation of the NF-κB pathway derives from restraining the generation of ROS. Lastly, there exist numerous disparities between rats and humans, thus rendering the animal models employed in this investigation inadequate in faithfully mirroring the pathogenesis of human ailments [38]. Therefore, further research is needed.

## Conclusion

In this work, we validated the protective effects of phillyrin on intervertebral disc degeneration through in vitro cell culture, exvivo, and in vivo experiments. Additionally, we explored and discovered the inhibitory effects of phillyrin on ROS production and the NF-kB pathway activation. In conclusion, our study proved that phillyrin prevents cell damage by reducing the generation of ROS, thereby alleviating ECM degradation and the NPCs apoptosis.

## Data Availability

No datasets were generated or analysed during the current study.
